# Advancements related to probiotics for preventing and treating recurrent respiratory tract infections in children

**DOI:** 10.3389/fped.2025.1508613

**Published:** 2025-02-06

**Authors:** Yali Zhang, Yingying Xu, Ling Hu, Xiaomei Wang

**Affiliations:** ^1^ Tianyou Hospital, Affiliated to Wuhan University of Science and Technology, Wuhan, Hubei, China; ^2^ Hubei Province Key Laboratory of Occupational Hazard Identification and Control, College of Medicine, Wuhan University of Science and Technology, Wuhan, Hubei, China

**Keywords:** recurrent respiratory tract infections, probiotics, children, prevention, health

## Abstract

Recurrent respiratory tract infections (RRTIs) are a common condition in pediatrics and significantly impact children's quality of life; however, their pathogenesis and contributing factors are not yet fully elucidated. Probiotics have recently emerged as promising agents for modulating intestinal microecology and have gained considerable attention in clinical research on preventing and treating RRTIs in children. This article provides an initial overview of the concept, classification, and mechanisms underlying probiotics. It emphasizes their beneficial effects on respiratory health by modulating intestinal microbial equilibrium, augmenting immune system functionality, and attenuating inflammatory responses. Subsequently, we examine existing research regarding the use of probiotics in pediatric RRTIs. Numerous clinical trials have unequivocally demonstrated that supplementing with probiotics can significantly reduce both the frequency and severity of RRTIs in children while also simultaneously decreasing antibiotic usage. However, there are ongoing controversies and challenges in current research concerning the influence of probiotic type, dosage, duration of use, and other factors on efficacy. Furthermore, variations have been observed across different studies. Additionally, it is crucial to further evaluate the safety and potential long-term side effects associated with probiotic use in children with RRTIs. In conclusion, we propose future research directions including conducting more high-quality randomized controlled trials to optimize application strategies for probiotics alongside other treatments while considering variations based on age and health conditions among pediatric populations. Finally, in summary although probiotics exhibit promising benefits in preventing and treating RRTIs in children; additional studies are necessary to refine their application strategies ensuring both safety and effectiveness.

## 1 Introduction

RRTIs are highly prevalent respiratory diseases affecting pediatric populations worldwide, particularly young children. According to international research data, approximately 10%–15% of children experience RRTIs at some point, representing about one-third of cases seen in primary pediatric care settings and accounting for between 8% and 18% of acute hospitalizations related to respiratory infections. Preschool-aged children are especially susceptible, experiencing an average annual occurrence rate ranging from six to ten viral colds (1). Globally, these infections claim the lives of approximately four million individuals each year while remaining the leading cause of mortality among those under six years old (2). Following the onset of RRTIs, common symptoms include fever and cough. If left untreated, they can lead to a decline in lung function among affected children, significantly impacting their health as well as growth and development (3). During early childhood, RRTIs often result in frequent medical consultations and emergency room visits while exerting a substantial influence on both affected children's quality of life and that of their families. Additionally, it frequently disrupts schooling activities (4). Enhanced resistance levels and concerns over treatment effectiveness often arise from the heavy reliance on antibiotics and antiviral drugs in conventional treatments for RRTIs. The inappropriate use of antibiotics has led to a significant increase in rates of bacterial resistance, resulting not only in elevated patient mortality but also imposing substantial economic pressure on healthcare systems (5).

Probiotics are commonly defined as living microorganisms that confer positive health effects on the host when present in sufficient quantities (6). In recent years, public attention towards probiotics has continued to rise due to the increasing recognition of their various beneficial effects in the field of health (7). According to statistics, the global probiotic market is projected to exceed $90 billion by 2026. Probiotics have been extensively used as pharmaceuticals, food additives or nutritional supplements for preventing and treating pediatric diseases (8). By modulating the cellular and humoral immune mechanisms, probiotics can enhance their immune defense capability (9). The research findings of evidence-based medicine suggest that oral probiotics effectively prevent respiratory tract infections and reduce recurrence rates in healthy children, without any observed adverse side effects (10, 11). Probiotics are highly esteemed for their diverse effects, particularly their antiviral properties. Recently, probiotics with antiviral capabilities have been incorporated into dairy products and fermented foods to assist in preventing viral infections (12). Evidence suggests that the utilization of probiotics can diminish the susceptibility to upper respiratory tract infections and facilitate the establishment of a robust microbiota environment in the upper respiratory tract that exhibits resistance against viral intrusion (13, 14).

The human gut microbiome represents a complex community of microorganisms residing within the gastrointestinal tract, encompassing an estimated range of 500–1,000 distinct bacterial species (15). Dysbiosis within this microbial ecosystem weakens host defense mechanisms against drug resistance and pathogenic bacteria colonization, increasing susceptibility to pathogens (15). Studies on human microbiota have revealed that intestinal flora can affect immune responses in multiple mucosal systems and facilitate systemic immune maturation (16), playing a crucial role in maintaining physiological equilibrium and influencing human health. Multiple studies (17, 18) have demonstrated a direct correlation between intestinal dysbiosis and impairment of the intestinal mucosal barrier integrity, resulting in an increased susceptibility to pulmonary infections. Recent investigations have extensively elucidated the link between imbalanced gut microbiota composition and RRTIs. The etiology of RRTIs is multifaceted. However, probiotics are widely acknowledged for their capacity to modulate both microecology and immune responses (19). These microorganisms can stimulate antigen-presenting cells (APCs), regulate cytokine secretion, and immunoglobulin production levels, while also reinforcing the protective layer on intestinal mucosal cells. Moreover, they exert influence over mucus generation while conferring resistance against invading pathogens or viruses (20). The objective of this review is to provide a comprehensive overview of current research advancements on probiotics in preventing and treating RRTIs in children. Additionally, it proposes several innovative strategies to address the limitations associated with probiotic therapies for RRTIs. This involves delving deeper into the underlying mechanisms, summarizing findings from clinical trials, addressing existing challenges, and identifying key areas for future research. By synthesizing findings from multiple studies, this review seeks to offer clinicians, researchers, and policymakers a multifaceted perspective that promotes evidence-based utilization of probiotics in pediatric healthcare while also highlighting areas requiring further investigation.

## 2 Correlation between RRTIs and intestinal flora in children

**Figure 1:**
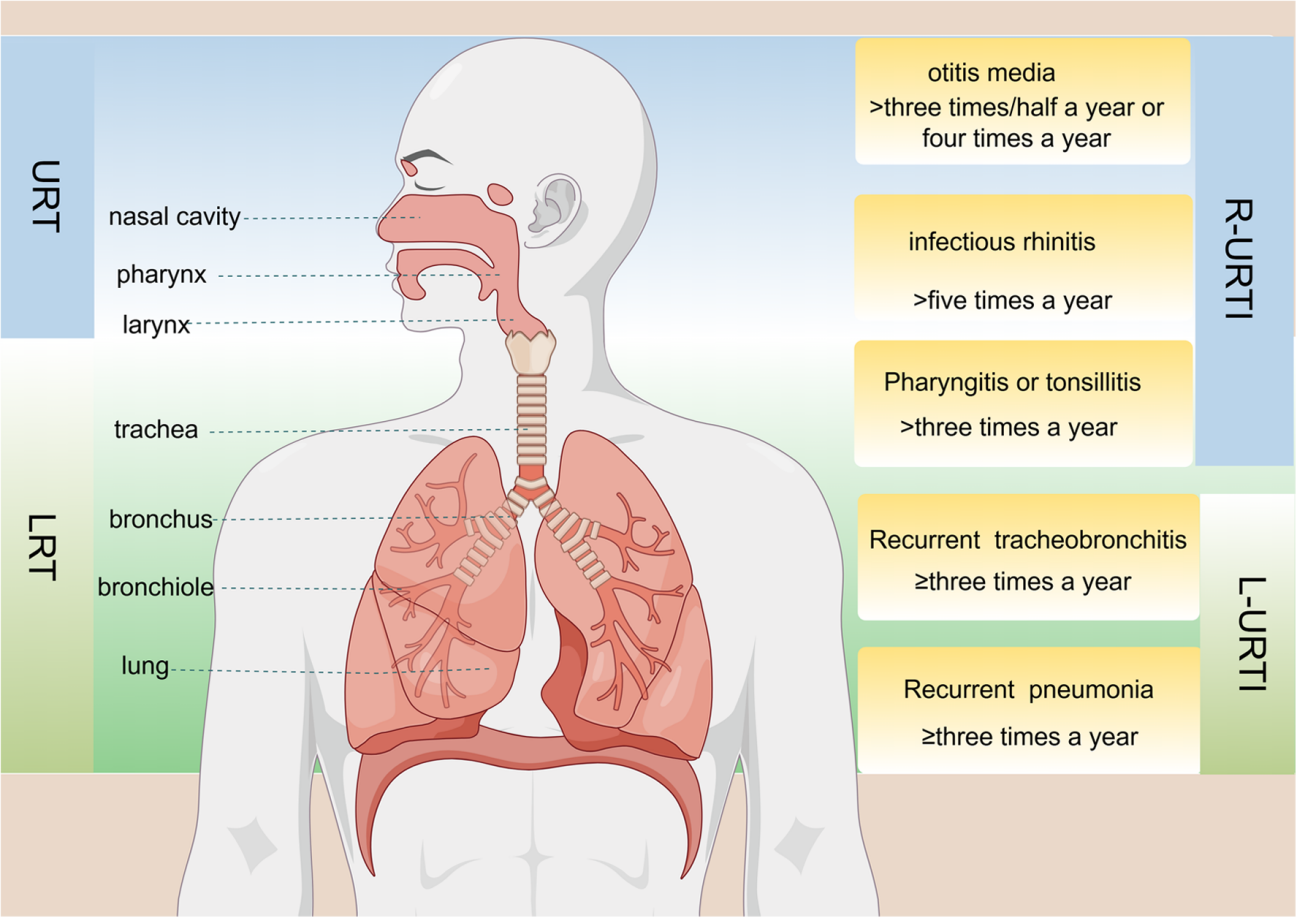
Anatomical structure of the respiratory tract and the definition of specific diseases associated with recurrent upper respiratory tract infections (R-URTIs) and recurrent lower respiratory tract infections (L-URTIs).

Currently, there is no global consensus on the diagnostic criteria for RRTIs in children. Different countries have varying definitions for RRTIs; however, the methods employed to diagnose them are consistent and based on infection frequency (Figure 1). Most scholars tend to define childhood RRTIs as eight or more documented respiratory infections per year in children under 3 years old, and six or more episodes in children over 3 years old (21, 22). RRTIs encompass both recurrent upper respiratory tract infections and recurrent lower respiratory tract infections (23). While there is no consensus on the exact definition of relapse, certain specific respiratory diseases have precise and unambiguous criteria for relapse. For instance, infectious rhinitis occurring more than five times per year is considered a recurrence in cases of recurrent upper respiratory tract infections, while acute otitis media recurring three times within six months or four times within twelve months also qualifies as a relapse (22). Recurrent lower respiratory tract infections are defined as bronchitis, bronchiolitis, or pneumonia occurring three or more times per year (24). In healthy individuals, a symbiotic and mutually regulated dynamic equilibrium exists between the microorganisms in the respiratory tract and intestine and their hosts, which is crucial for maintaining normal physiological functions. However, when pathogenic microorganisms invade the human body, this delicate microbial balance can be disrupted, resulting in the development of pathological conditions (25). Due to their less responsive immature immune systems towards environmental pathogens (26), children often encounter respiratory tract infections. The intestine functions as a pivotal immunological organ crucial for the maturation and differentiation of immune cells (27). The intestinal microbiota can modulate the cells of the intestinal epithelium, facilitating immune tissue development associated with the intestinal mucosa and activating receptors on immune cells (28). It not only plays a local regulatory role in the mucosal immune system but also has a pivotal regulatory role in cell-mediated systemic immune response (29). In addition to aiding nutrient absorption, gut microbiota defends against infections by competing for resources with pathogens or secreting antibacterial substances that eradicate pathogens (30). The gut-lung axis refers to bidirectional communication established between intestinal microorganisms and their metabolites, which regulate or exchange information between intestinal and lung tissues via circulation through blood and lymphatic systems (31). Previous studies have demonstrated that this regulatory network influences respiratory diseases through its impact on gut microbiota (32). Additionally, histoembryological evidence confirms that both the intestine and respiratory tract originate from common embryonic tissue known as the foregut of the gastrum, with their mucosal inner walls being continuous (33). Due to the thinner intestinal wall, higher permeability, and weaker barrier function in children with RRTIs, allergens such as toxins and incompletely digested products may enter the body through the intestine and subsequently reach the lungs via blood circulation. Several studies have compared populations of *Bifidobacterium* and *Lactobacillus* in the gut of children with RRTIs to healthy children, revealing a significant reduction. This dysbiosis in intestinal microbiota leads to decreased levels of IgA-dominated immunoglobulins (34).

## 3 The mechanism of probiotics intervention on RRTIs in children

**Figure 2:**
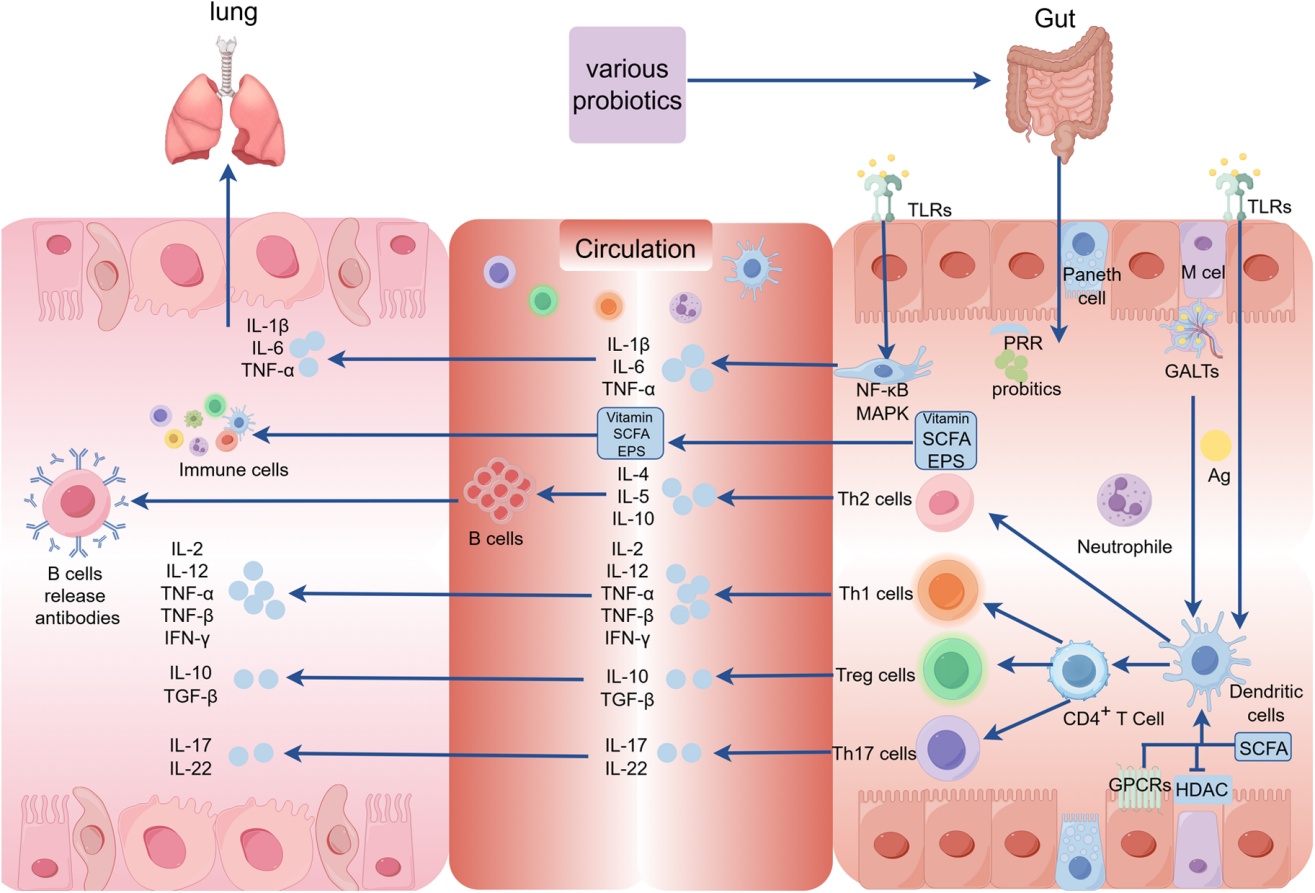
The mechanism underlying the intervention of probiotics on RRTIs in children. (GALTs, gut-associated lymphoid tissue; PRR, pattern recognition receptors; MAPK, mitogen-activated protein kinase; GPCRs, G protein-coupled receptors; HDAC, histone deacetylases; SCFA, short-chain fatty acids; TLRs, toll-like receptors; Ag, antige; EPS, exopolysaccharides).

Probiotics are defined as “living microorganisms that confer health benefits to the host when administered in sufficient quantities” (35). *Lactobacillus and Bifidobacteria*, which are widely used in the field of probiotics (36), secrete various beneficial compounds like vitamins, short-chain fatty acids (SCFAs), bacteriocins, and exopolysaccharides (EPS) that contribute to their positive effects (37). Most children develop RRTIs due to their immature immune system during development, and typically there is no underlying disease present during this period (23). Certain probiotics strains play a crucial role in preventing and treating respiratory diseases by inhibiting pathogen growth and harmful bacteria, producing antimicrobial substances, enhancing the defense function of the epithelial cell barrier, and importantly regulating immune system activity (22), thereby impacting both innate and adaptive immunity in the host (Figure 2). Recent studies have unveiled a correlation between dysbiosis of intestinal flora and RRTIs. For instance, strains such as *Lactobacillus rhamnosus* and *Escherichia coli* Nissle 1917 can activate pattern recognition receptors (PRRs) on epithelial cells through microbially associated molecular patterns to regulate tight junctions and adhesion junctions, thereby preserving the integrity of the epithelial barrier (38). The probiotics inhibit the proliferation of pathogenic bacteria by competing for adhesion sites on epithelial cells or depleting their essential nutrients, while simultaneously secreting antibacterial substances to establish an unfavorable microenvironment for pathogen survival (39). Additionally, probiotic metabolites like SCFAs modulate the differentiation of immune cells and control excessive immune responses by interacting with G protein-coupled receptors (GPCRs) and inhibiting histone deacetylases (HDACs), thereby playing a crucial role in immune response, inflammation, and lung disease development (40). Moreover, probiotics can exhibit antiviral effects through direct interaction with viruses or the generation of antiviral metabolites while stimulating the host's immune system response (41). Gut-associated lymphoid tissue (GALTs) is a vital part of the peripheral immune system that houses around 80% of active immune cells. Probiotics have the potential to enhance systemic immunity by modulating GALTs' response, indirectly strengthening the respiratory tract's defense capabilities (42). Upon encountering intestinal microbiota, infectious pathogens, as well as ingested or inhaled antigens, GALTs facilitate frequent interactions between immune cells and these antigens, thus triggering an ongoing immune response that encompasses germ-center (GC) formation within intestinal microbiota and subsequent responses to pathogen invasion (43). The specific mechanism involves the encapsulation of GALTs by microfold (M) cells, facilitating their targeted delivery to dendritic cells (DCs) and subsequent antigen exposure, thereby initiating immune responses from T and B cells in the mucosa (44). Furthermore, probiotics mediate the innate immune response through PRRs, particularly toll-like receptors (TLRs), which recognize and bind to pathogen-associated molecular patterns (PAMPs) on the surface of pathogenic microorganisms. This interaction triggers the activation of signaling pathways such as nuclear factor-κB (NF-κB) and mitogen-activated protein kinase (MAPK) (45, 46). As a result, this process modulates the secretion of pro-inflammatory cytokines including tumor necrosis factor-α (TNF-α), interleukin-6 (IL-6), and interleukin-1β (IL-1β), thereby regulating host-pathogen interactions and influencing the immune and inflammatory responses. *Lactococcus lactis* can initiate signal transduction in DCs through the TLR2, TLR3, and TLR9 pathways, thereby promoting DC maturation, enhancing Th1 cell differentiation, and upregulating the expression of IL-12, IFN-γ, and TNF-α (47). Additionally, probiotics have been shown to induce clonal expansion of IgA-producing B cells by elevating IL-6 levels in a TLR2-dependent manner (37). Therefore, they serve as a preventive and therapeutic strategy against respiratory infections.

## 4 Clinical application of probiotics for preventing and treating RRTIs in children

### 4.1 Clinical application of individual probiotics

*Lactobacillus rhamnosus* GG (LGG), isolated in 1983 by Gorbach and Goldin, is currently the most widely utilized probiotic due to its resistance to gastric acid and bile attack. As a probiotic strain, LGG confers gastrointestinal benefits (48) by regulating the microbial balance in the gut (49). It reduces harmful bacteria like *Bacteroides* and *Proteus* while increasing beneficial bacteria such as *Lactobacillus*, *Bifidobacterium*, and *Butyricococcus* (50–52). Additionally, LGG modulates the host's immune system with antiviral and antibacterial properties that help prevent and treat specific infections. Research has demonstrated that LGG safeguards intestinal epithelial cells from destruction by triggering an inflammatory response and activating macrophages (53, 54). Despite the manifold health benefits demonstrated by LGG, the findings exhibit some inconsistencies and potential interindividual variations in probiotic efficacy may exist.

The effectiveness of LGG has been proven in multiple studies for preventing and treating respiratory infections among children. In a randomized trial involving 281 children attending daycare centers, LGG significantly decreased upper respiratory tract infections and reduced the risk of respiratory tract infections lasting more than 3 days while also shortening the duration of respiratory symptoms. However, it is noteworthy that there was no significant effect observed on the risk of lower respiratory tract infections associated with LGG intake. These findings suggest that LGG may serve as an effective approach to mitigate upper respiratory tract infection risks among children attending daycare centers (55). Furthermore, based on four randomized controlled trials encompassing 1,805 participants' data analysis results indicate that LGG also diminishes acute otitis media (AOM) incidence rates along with antibiotic usage (56). Moreover, subgroup analysis focusing on children over 1 year old further confirms that LGG significantly decreases overall risks of respiratory infections (56). Another study showed that LGG significantly reduced the duration of respiratory tract infections (RTIs) (57). Additionally, LGG (ATCC 53103) can effectively decrease the frequency of rhinovirus-induced RTIs, highlighting its positive impact on reducing RTI incidence caused by rhinovirus infection in preterm infants during their first year of life (58). While LGG has demonstrated efficacy in ameliorating respiratory symptoms, there is insufficient statistical evidence supporting its antiviral activity and ability to mitigate associated manifestations. Rhinovirus, respiratory syncytial virus (RSV), and parainfluenza virus type 1 stand as the primary causative agents of pediatric respiratory ailments. In a 28-week randomized controlled trial with double-blinding and placebo control measures, LGG notably reduced monthly days with respiratory symptoms among children. However, it did not significantly influence the detection rate or severity of viral pathogens when present. While displaying potential for effectively alleviating respiratory distresses through symptomatic relief mechanisms alone without directly inhibiting viral infections (59). Overall, LGG may effectively reduce the risk of upper respiratory infections in children. Nevertheless, its effectiveness might exhibit interindividual variability. In a randomized study involving 619 participants aged 2–6 years and lasting for 16 weeks, it was observed that consumption of LGG DSM 33156 ameliorated symptoms during upper respiratory infections (URTIs) in children. But it is regrettable that the study failed to provide substantial evidence supporting a significant reduction in the incidence of URTIs (60).

Bifidobacterium is a group of beneficial bacteria commonly found in the human gut, especially in infant intestines. However, their abundance and species diversity tend to decline with age (61). The Gram-positive bacteria belonging to the family *Bifidobacteriaceae* are classified as members of *Bifidobacteria*. These bacteria are typically anaerobic but can occasionally tolerate oxygen. They lack spore-forming ability and exhibit various morphological forms (61). Due to its immunomodulatory function on the neonatal immune system, *Bifidobacterium* has garnered significant research attention (62–64). Studies have demonstrated that *Bifidobacteria* play a crucial preventive role in maintaining a healthy intestinal microbiota by regulating probiotic metabolism, promoting intestinal motility, adhering to and degrading harmful substances, as well as enhancing host immune function (65).

*Bifidobacterium longum*, a member of the *Bifidobacterium* genus, holds a dominant position in infants' intestinal microbiota and plays an indispensable role in their healthy growth and development. It can modulate the Th1/Th2 immune system balance to prevent associated diseases (66). A study conducted on Malaysian preschoolers demonstrated that *Bifidobacterium elongatus* BB536 can significantly enhance the abundance of anti-inflammatory and immunomodulatory fecal bacteria, potentially protecting against upper respiratory diseases by regulating gut microbiota (67).

*Lactobacillus,* a subspecies of *Bifidobacterium* animalis, has gained significant attention in the field of neonatal intestinal microbiota research due to its remarkable safety profile and multitude of positive effects on intestinal diseases (68, 69). In a double-blind study by Taipale et al., it was observed that early administration of BB-12 probiotics in infants reduced the risk of early infection and respiratory infections (70). However, other studies have indicated that *Bifidobacterium* animalis *subsp. lactis* may not exhibit efficacy in preventing common infections among hospitalized children, potentially attributed to lower-than-expected incidence rates of nosocomial infections (71).

*Streptococcus salivarius*, a constituent of the human oral microbiota, has been extensively investigated for its potential oral health benefits, particularly in the preventing and alleviating sore throats. The secretion of *Lactobacillus acidophilus* and salivary peroxidase by *Streptococcus salivarius* help maintain an optimal acid-base balance in the oral cavity and inhibits the proliferation of pathogenic bacteria, reducing bacterial load and minimizing sore throat occurrence. Research has demonstrated that *Streptococcus salivarius* exerts immune regulatory effects that restrict viral infectivity (72).

*Streptococcus salivarius* demonstrates exceptional oral adhesion capabilities while also producing bacteriocin-like inhibitors and possessing immunomodulatory properties. It has been employed for managing pharyngitis, tonsillitis, and otitis media (73). Studies have shown that administering prophylactic doses of *S. salivarius* K12 (*Bactoblis*®) to children who are prone to recurrent oral streptococcal infections can significantly reduce both bacterial/viral infection rates as well as the need for antibiotics or antipyretics (74). The clinical studies conducted by Wescombe, P.A., et al. have also shown the therapeutic potential of *Streptococcus salivarius* K12 strain in the treatment of streptococcal sore throat (75). Furthermore, *S. salivariu*s, a non-pathogenic species and an integral member of the normal oral microbiome, has exhibited efficacy in reducing recurrent colonization by major pathogens in the URT. The effectiveness of intranasal administration of *Streptococcus sialus* 24SMB in reducing the incidence and frequency of antibiotic usage for recurrent acute otitis media in children aged 1–5 years was demonstrated through a prospective, randomized, double-blind, placebo-controlled design (76). Interestingly, clinical trials have indicated that *S salivarius* K12 does not exhibit a reduction in the occurrence of AOM in children (77).

*Lactobacillus casei*, a prevalent strain of the genus *Lactobacillus*, is commonly found in various natural sources including dairy products, vegetables, and meat. It exerts its immunomodulatory effects by enzymatically hydrolyzing casein into immunogenic peptides (78). In a 12-week clinical trial involving 1,003 Vietnamese children aged 3–5 years, daily consumption of fermented dairy products containing *Lactobacillus casei strain Shirota* (LcS) effectively reduced the incidence of constipation and acute respiratory infections (ARIs) among these children (79).

*Bacillus coagulans* is a highly stable probiotic strain with a long history of safe use in food and has been granted Generally Recognized as Safe (GRAS) status by the U.S. Food and Drug Administration, making it an ideal candidate for food and pharmaceutical preparations. It exhibits various beneficial roles such as regulating microbiome composition, immune response, and metabolism (80). GanedenBC30, scientifically known as *Bacillus coagulans*, is extensively used in health products and food items to promote gastrointestinal well-being and enhance immune system functionality. The study conducted by M.A. et al. demonstrated that the administration of probiotic GanedenBC30 (*Bacillus coagulans* GBI-30, 6,086 strain) had positive effects on the frequency, duration, and severity of symptoms associated with URTIs, as well as reducing the occurrence of distension events in school-age children (81).

### 4.2 Clinical application of complex probiotics

Complex probiotics, also known as multi-bacterial probiotics, encompass preparations containing two or more distinct strains of probiotics. These formulations possess the ability to modulate the composition and diversity of intestinal flora, safeguard intestinal barrier function, and mitigate inflammation and intestinal damage. Using complex probiotics can address drug resistance arising from excessive use of a single strain. Previous studies have demonstrated that complex probiotics exhibit superior efficacy in maintaining intestinal health and regulating immune function compared to individual strains alone (82).

Complex probiotics have demonstrated potential positive effects in the prevention and treatment of RRTIs. Multiple studies indicated that specific combinations of probiotics can balance gut microbes and enhance immune system function, thereby reducing the frequency and severity of RRTIs. For example, probiotic formulations containing *Lactobacillus* BB-12 and *Enterococcal Streptococcus faecalis* L3 significantly increase salivary IgA levels and mitigate the risk of URTIs in healthy children (83). For children with recurrent respiratory infections, oral bifidobacteria quadruplex vaccine tablets containing *Bifidobacteria infantis, Lactobacillus acidophilus, Enterococcus faecalis* and *Bacillus cereus* not only enhanced the abundance of *Bifidobacteria* and *Lactobacilli* but also significantly reduced the average annual frequency of acute RTIs and antibiotic usage (34). Clinical trials show that probiotic sprays containing *Streptococcus salivarius 24SMB* and *Streptococcus oralis* 89a exhibited efficacy in preventing URTIs among pediatric patients (84). Additionally, they demonstrated a significant reduction in both the frequency of Group A beta-hemolytic *Streptococcus* (GABHS) infection episodes and antibiotic usage (84). Another study reported that *Streptococcus salivarius 24SMB* and *Streptococcus oralis* 89a effectively alleviated symptoms associated with recurrent respiratory infections in children (85). Furthermore, Campanella et al. provided further validation regarding the potential benefits and safety profile of probiotics for reducing the incidence of RTIs among pediatric populations (86). The combination of *Lactobacillus reuteri* ATCC PTA 5289 and *Lactobacillus reuteri* DSM 17938 has also demonstrated efficacy in alleviating symptoms associated with pharyngitis or tonsillitis in pediatric patients (87). In a clinical study, it was demonstrated that the preschool doses comprised *Lactobacillus acidophilus* CUL21 (NCIMB 30156), *Lactobacillus acidophilus* CUL60 (NCIMB 30157), *Bifidobacterium bifidum* CUL20 (NCIMB 30153), and *Bifidobacterium animalis subsp. lactis* CUL34 (NCIMB 30172) in Lab4 probiotic chewable tablets, along with 50 mg of vitamin C, which were effective in preventing URTIs and reducing the use of antibiotics (88). However, it should be noted that not all probiotics are equally effective against RTIs. The probiotic capsules containing *Lactobacillus acidophilus* CUL60, *Lactobacillus acidophilus* CUL21, *Bifidobacterium bifidum* CUL20, and *Bifidobacterium animalis ssp. lactate* were also found to have no significant impact on reducing the severity of symptoms associated with pharyngitis (89). Furthermore, a randomized controlled trial conducted by Santamaria et al. found no significant difference in respiratory tract infections or disease-free days when using *Bifidobacterium* mixtures (*B. longum* BB536, *B. infantis* M-63, *B. breve* M-16V) (90). In a Finnish double-blind, placebo-controlled trial, children aged 10 months to 6 years who were at high risk for OM received a daily probiotic capsule containing *Lactobacillus rhamnosus* GG, *Bifidobacterium breve* 99, and *Propionibacterium freudenreichii* JS. The study concluded that the probiotics did not significantly prevent AOM or reduce nasopharyngeal colonization by otitis media pathogens in these susceptible children (91).

In conclusion, although probiotics have demonstrated some efficacy in the prevention and treatment of RRTIs in children, their effectiveness may vary depending on the specific combination of strains and individual variations (Table 1). Therefore, meticulous selection of an appropriate probiotic product and customization of treatment for each individual is imperative to optimize efficacy.

**Table 1:** Clinical application of probiotics in the prevention and treatment of RRTIs in children.

Probiotics	Subjects	Taking instructions	Efficacy	Ref
*Lactobacillus rhamnosus*	Children in the day care center	Oral administration	Reducing the URTIs	(55)
	Children	Oral administration	Reduce the AOM	(56)
	Children over 1 year of age	Oral administration	Reduce the risk of overall RTIs	(56)
	Children in the day care center	Oral administration	Reduce the duration of RTIs	(57)
	Preterm infants in the first year of life	Oral administration	Reduce RTIs caused by rhinovirus	(58)
	Children	Oral administration	Reduce respiratory symptoms	(59)
	Children aged 2–6 years	Oral administration	Fail to reduce in the incidence of URTIs	(60)
*Bifidobacterium longum* BB536	Preschool children	Oral administration	Prevention of URTIs	(67)
*Bifidobacterium animalis* BB-12	Early Infancy	Oral administration	Reduce the RTIs	(70)
*Bifidobacterium animalis subsp. lactis*	Hospitalized children	Oral administration	Fail to shorten the duration of RIs and prevent infection	(71)
*Streptococcus salivarius* K12	Children at risk for recurrent oral streptococcal infection	Oral administration	Reduce the frequency of streptococcal and viral infections	(74)
	Children	Oral administration	Treatment of streptococcal sore throat	(75)
*Streptococcus sialus* 24SMB	Children aged 1–5 years	Nasal spray	Prevent the AOM	(76)
*Streptococcus salivarius* K12	Children aged 1–6 years	Oral administration	Fail to reduce the AOM	(77)
*Lactobacillus case*i	Children aged 3–5 years	Oral administration	Reduce the ARIs	(79)
*Bacillus coagulans* GBI-30	School age children	Oral administration	Reduce the frequency and symptoms of URTIs	(81)
*Lactobacillus* BB-12 *Enterococcal Streptococcus faecalis* L3	Children	Oral administration	Prevent the URTIs	(83)
*Bifidobacteria infantis* *Lactobacillus acidophilus* *Enterococcus faecalis* *Bacillus cereus*	Children with RTIs	Oral administration	Reduce the acute RTIs	(34)
*Streptococcus salivarius* 24SMB *Streptococcus oralis* 89a	Children	Nasal spray	Prevent recurrent URTIs	(84)
	Children	Nasal spray	Reduce the frequency of GABHS infection	(84)
	Children with RRTIs	Nasal spray	Relieve the symptoms	(85)
*Lactobacillus reuteri* ATCC PTA 5289 *Lactobacillus reuteri* DSM 17938	Children 6 months to 5 years of age with pharyngitis or tonsillitis	Oral administration	Relieve the symptoms of pharyngitis or tonsillitis	(87)
*Lactobacillus acidophilus* CUL21 *Lactobacillus acidophilus* CUL60 *Bifidobacterium bifidum* CUL20 Bifidobacterium lactate CUL34	Children aged 3–6 years	Oral administration	Prevent the URTIs Reduce the use of antibiotics	(88)
*Lactobacillus acidophilus* CUL60 *Lactobacillus acidophilus* CUL21 *Bifidobacterium bifidum* CUL20 *Bifidobacterium animalis ssp. lactate*	Children aged ≥3 years with pharyngitis	Oral administration	Fail to reduce pharyngitis symptoms	(89)
*Bifidobacterium longum* BB536 *Bifidobacteria infantis* M-63 *Bifidobacterium breve* M-16V	RRTIs children aged 3–6 years	Oral administration	Fail to reduce the frequency of RTIs	(90)
*Lactobacillus rhamnosus* GG *Bifidobacterium breve* 99 *Propionibacterium freudenreichii* JS	Children aged 10 months to 6 years	Oral administration	Fail to prevent the occurrence of AOM	(91)

URTIs, upper respiratory infections; RRTIs, recurrent respiratory tract infections; AOM, acute otitis media; ARIs, acute respiratory infections; GABHS, group A beta-hemolytic *Streptococcus*.

## 5 Safety and adverse reactions of probiotics

Probiotics, which are a consortium of living bacteria, exert beneficial effects on the human body by modulating the gut microbiome, enhancing immune function, and improving digestive health. Although numerous probiotic products have demonstrated safety (92), their usage may also lead to certain adverse reactions. Currently, there is no globally unified standard for evaluating probiotics due to ongoing advancements in safety assessments across different countries. The utilization of probiotics may pose several potential risks and adverse effects. For example, some clinical reports have indicated that probiotics can lead to gastrointestinal side effects such as diarrhea and bloating (93). In addition, it is imperative to acknowledge the potential for horizontal gene transfer of antibiotic resistance genes from probiotic strains to other microorganisms. In 1998, viable probiotic bacteria were identified as donors in the conjugative transfer of antibiotic resistance (AR) genes, a phenomenon subsequently corroborated by additional studies confirming the intestinal transfer capability of AR genes (94). Moreover, AR gene transfer can also occur through transformation via free DNA or phage-mediated transduction (95). As highlighted in the preceding clinical application section, probiotics generally have exhibited a relatively favorable safety profile among healthy pediatric populations. However, it has been reported that certain probiotic strains may act as opportunistic pathogens in immunocompromised individuals, potentially leading to adverse effects such as life-threatening conditions like pneumonia (96), endocarditis (97), and sepsis (98). To ensure the safe application of probiotics, it is crucial to perform whole genome sequencing of probiotic strains. This method not only facilitates strain-level identification but also enables the detection of virulence factors, pathogenicity determinants, and AR genes, thereby enhancing the safety and efficacy of probiotic use (95).

## 6 Summary and prospect

The present review comprehensively analyzes recent advancements in using probiotics for preventing and treating RRTIs in pediatric patients. Multiple studies have demonstrated that probiotics exert a favorable influence on reducing the incidence of RRTIs and alleviating symptoms by modulating the gut microbiome, enhancing immune system functionality, attenuating inflammatory response, and other underlying mechanisms. In terms of prevention, multiple randomized controlled trials have consistently demonstrated the significant efficacy of specific strains of probiotic supplementation in reducing the incidence and duration of RRTIs in children. Moreover, probiotics have also been found to effectively decrease antibiotic usage frequency, thereby mitigating risks associated with adverse reactions and resistance. Regarding treatment, although available evidence is not as robust as that for prevention studies, preliminary research suggests that probiotics may expedite recovery from respiratory infections by enhancing host immune function. However, it should be noted that considerable variation exists in the efficacy of different strains and combinations necessitating further high-quality investigations to determine optimal treatment options. Despite showing promise in preventing and treating RRTIs in children, several challenges and unresolved issues still persist. For example, further investigation is required regarding aspects such as optimal dosage, duration, strain selection, and suitability for specific pediatric populations concerning probiotics. Moreover, significant gaps persist in understanding the long-term effects of probiotics on pediatric populations, particularly in children with chronic or recurrent conditions. When administering probiotics, it is imperative to conduct a comprehensive evaluation of individual differences, lifestyle factors, genetic backgrounds, potential risks, and long-term outcomes to ensure both the safety and efficacy of these interventions. The significance of whole-genome sequencing as an indispensable tool for evaluating the safety of probiotic strains cannot be overstated. It not only enhances strain identification for production tracing and infection investigation but also facilitates the classification and assessment of associated risks. Additionally, it enables the detection of potential virulence, pathogenicity, or antibiotic resistance genes within genomes. Probiotic formulations must ensure safety regarding purity, potency, and composition through rigorous testing protocols to prevent contamination, especially with live organisms. Stricter testing measures are imperative for products intended for vulnerable populations. In conclusion, while probiotics hold promising prospects in preventing and treating RTIs in children, their efficacy and safety necessitate verification through additional clinical trials. Future research ought to concentrate on identifying the optimal combination and usage strategies for probiotics, as well as exploring their integration with other interventions (e.g., vaccination, nutritional support) to provide more comprehensive approaches towards safeguarding children's health.
